# Proteomic analysis revealed T cell hyporesponsiveness induced by *Haemonchus contortus* excretory and secretory proteins

**DOI:** 10.1186/s13567-020-00790-0

**Published:** 2020-05-13

**Authors:** Mingmin Lu, Xiaowei Tian, Zhang Yang, Wenjuan Wang, Ai-Ling Tian, Charles Li, Ruofeng Yan, Lixin Xu, Xiaokai Song, Xiangrui Li

**Affiliations:** 1 MOE Joint International Research Laboratory of Animal Health and Food Safety, College of Veterinary Medicine, Nanjing Agricultural University, Nanjing, 210095 Jiangsu China; 2 State Key Laboratory of Veterinary Etiological Biology, Lanzhou Veterinary Research Institute, Chinese Academy of Agricultural Sciences, Lanzhou, 730046 Gansu China; 3 Animal Biosciences and Biotechnology Laboratory, Beltsville Agricultural Research Center, Agricultural Research Service, U.S. Department of Agriculture, Beltsville, MD 20705 USA

## Abstract

*Haemonchus contortus* has evolved highly integrated and sophisticated mechanisms to promote coexistence with hosts. The excretory-secretory (ES) products generated by this parasite contribute to the regulation of the host immune response to facilitate immune evasion and induce chronicity, but the proteins responsible for this process and the exact cellular mechanisms have yet to be defined. In this study, we identified 114 *H. contortus* ES proteins (HcESPs) interacting with host T cells and 15 T cell binding receptors via co-immunoprecipitation and shotgun liquid chromatography-tandem mass spectrometry analysis. Based on bioinformatics analysis, we demonstrated that HcESPs could inhibit T cell viability, induce cell apoptosis, suppress T cell proliferation and cause cell cycle arrest. Furthermore, the stimulation of HcESPs exerted critical control effects on T cell cytokine production profiles, predominantly promoting the secretion of interleukin (IL)-10, IL-17A and transforming growth factor-β1 and inhibiting IL-2, IL-4 and interferon-γ production. Collectively, these findings may provide insights into the interaction between ES proteins and key host effector cells, enhancing our understanding of the molecular mechanism underlying parasite immune evasion and providing new clues for novel vaccine development.

## Introduction

Epidemiological data suggest that more than one billion people worldwide, as well as numerous groups of livestock, are infected with at least one species of gastrointestinal (GI) nematode [1]. These parasitic species have evolved sophisticated and highly integrated mechanisms to reside in the GI tract of the hosts [2]. GI nematodes can release certain factors, generally termed excretory-secretory (ES) products or proteins, by actively exporting or passively diffusing into the host environment to ensure survival [2, 3]. To date, the investigation of nematode ES proteins has been incorporated into taxonomic composition analysis, immunodiagnostic applications, and vaccine development [4]. Importantly, increasing attention has been paid to the immunomodulatory properties of ES proteins, with multitudinous findings demonstrating the selective immunosuppressive or regulatory effects of certain nematode products on host immune cells [5].

The barber’s pole worm, *Haemonchus contortus*, is a voraciously blood-feeding and highly pathogenic GI nematode inhabiting the abomasum of small ruminants (mainly sheep and goats) [6]. Given its widespread occurrence and substantial morbidity among affected animals, *haemonecrosis* is one of the most economically important parasite diseases, representing a major constraint on the livestock industry worldwide, especially in tropical, subtropical and warm climatic zones [7]. *H. contortus* is transmitted via a complex life cycle involving three free-living larval stages and two parasitic stages. After oral ingestion by the host in contaminated pastures, the infective third-stage larvae (L3) moult into the parasitic fourth-stage larvae (L4) via an exsheathment process triggered by the gastric acidic environment and then develop into adults, causing severe pathology and normally inducing chronicity [8].

Unlike the defined rapid and delayed rejection of L3 and IgA-induced hypobiosis toward L4 feeding, little is known about the exact molecular basis of host protective mechanisms against adult worm-mediated damage [9]. Due to anthelmintic resistance and the increasing demands for drug-free animal production [10], a better understanding of the mechanisms by which adult worms regulate host immune responses to promote coexistence with hosts may contribute to the exploitation of novel control strategies against *H. contortus* infection. Importantly, accumulating evidence has revealed that an array of adult *H. contortus* ES proteins (HcESPs), for example, Hco-gal-m/f [11], HcSTP-1 [12], Miro-1 [13], and Hc-AK [14], contribute to the facilitation of immune evasion by suppressing the proliferation of host peripheral blood mononuclear cells (PBMCs) and the production of protective cytokines.

Similar to other GI nematodes, host cellular immunity against *H. contortus* infection is associated with the establishment of a type 2 immune response characterized by the secretion of interleukin (IL)-4, IL-5 and IL-13, as well as the development of a Th1-type immune response related to chronic infections [9]. As the regulators and the regulated at the host-parasite interface, T cells play pivotal roles against GI nematode infections. However, immunosuppressed hosts cannot generate persistent and effective anti-nematode immunity clinically due to the impairment of T cell functions. For instance, CD4^+^ Th2 responses were notably inhibited by myeloid-derived suppressor cells induced by primary *Heligmosomoides polygyrus* (HP) infection [15], and HP infection could also block T cell activation by promoting P-glycoprotein activity [16]. Moreover, recent studies demonstrated that ES products derived from GI nematodes contributed to suppressing host T cell responses, as exemplified by the inhibition of CD4^+^ and CD8^+^ T cell proliferation induced by *Ancylostoma caninum* and *Toxocara canis* ES proteins [17]. However, the exact role of T cells as putative key effector cells in *H. contortus* infection is still poorly understood, and the exact molecular basis of the regulation between T cells and HcESPs remains to be elucidated. Based on the stage-specific binding of HcESPs to host PBMCs in vivo [18] and the immunosuppressive effects of HcESPs on PBMCs in vitro [19], the aim of this study was to characterize the interactions between HcESPs and host T cells, as well as to elucidate the immunomodulatory potential of HcESPs in the T cell-mediated immune response. To our knowledge, this is the first proteomics-guided comprehensive investigation of the relevance of HcESPs to host key effector cells.

## Materials and methods

### Ethics statement

All experimental protocols were reviewed and approved by the Science and Technology Agency of Jiangsu Province (Approval No. SYXK (SU) 2010–0005). All animal experiments were performed in strict compliance with the guidelines of the Animal Welfare Council of China. All efforts were made to minimize the suffering of animals, and daily health checks were performed throughout the experiments.

### Parasite, animals and cells

The *H. contortus* strain was preserved in the laboratory of veterinary parasitology at Nanjing Agricultural University. Worms were maintained and propagated by serial passage in nematode-free goats (5–6 months old). The recovery procedures for the eggs and L3 of *H. contortus* were performed as previously described [20].

Sprague–Dawley (SD) rats (body weight ~150 g) were purchased from the Experimental Animal Center of Jiangsu, China (quality certificate: SCXK 2008-0004) and were raised in a sterilized room with free access to sterilized food and water.

Local crossbred and healthy goats (5–6 months of age) were reared in individually ventilated cages to prevent accidental infection with nematodes. They were fed with hay and whole shelled corn and provided water ad libitum in pens. Peripheral venous blood samples were obtained by venipuncture from these goats, and the isolation of goat PBMCs was conducted as previously described [21]. Total T cells were sorted from goat PBMCs by a magnetic-activated cell sorting (MACS) system (Miltenyi Biotech, Auburn, CA, USA) as described elsewhere [22]. Briefly, freshly isolated PBMCs were resuspended to a density of 1 × 10^6^ cells/mL in phosphate-buffered saline containing 2 mM EDTA and 0.5% bovine serum albumin (BSA, Sigma, St. Louis, MO, USA). Then, 1 × 10^6^ PBMCs in 100 µL of staining buffer were incubated with 10 μL of mouse anti-bovine CD2 primary antibody (Bio-Rad Inc, Kidlington, UK), which was cross-reacted with goat T cells at room temperature for 30 min. After two washes, 1 × 10^7^ total cells in 100 µL of staining buffer were labelled with 10 μL of anti-FITC MicroBeads (Miltenyi Biotec) at room temperature for 15 min. Subsequently, the cell suspension was loaded on a MACS MS Column (Miltenyi Biotec) placed in the magnetic field of the MACS Separator (Miltenyi Biotec). The magnetically labelled T cells were retained in the column, while the unlabelled cells passed through the column. After removing the column from the MACS Separator, T cells were eluted as the positively selected cell fraction. The T cells were then resuspended to a density of 1 × 10^6^ cells/mL in RPMI 1640 (Gibco, Grand Island, NY, USA) containing 100 U/mL penicillin and 100 mg/mL streptomycin (Gibco) and 10% heat-inactivated foetal calf serum (FCS, Gibco). The viability of the T cells was > 95%, as assessed by the trypan blue exclusion test. The purity of isolated T cells was above 95%, as measured by flow cytometry. Three biological replicates (three goats) were used in each experiment.

### Collection of HcESPs and generation of polyclonal antibodies

HcESP collection was performed following standard procedures as previously described [18]. Briefly, adult worms were obtained from the abomasum of experimentally infected goats, washed with PBS containing 1% penicillin/streptomycin (Gibco) five times, seeded into 6-well culture plates (100 worms per well), and cultured in 1% PS/RPMI-1640 (Gibco) at 37 °C for 24 h with worm viability assessment and medium exchange every 6 h. The supernatant containing HcESPs was harvested, pooled, centrifuged, filtered, and concentrated with an Amicon Ultra centrifugal tube with a 3-kD molecular weight cutoff (Merck Millipore, Bedford, MA, USA). HcESP concentrations were measured by the Bradford assay, and HcESP samples were stored at −80 °C until further analysis. Five biological replicates (five goats) were used for HcESP collection.

To generate polyclonal antibodies against HcESPs, 300 µg of HcESPs blended with Freund’s complete adjuvant was injected subcutaneously into SD rats for primary immunization. SD rats were later boosted four times with the same dose of HcESPs mixed with Freund’s incomplete adjuvant at 2-week intervals. One week after the last injection, rat sera containing specific anti-HcESP antibodies were collected and stored at −80 °C for later use.

### Immunoblot analysis

HcESP samples were resolved on 12% SDS-PAGE gels and then electrotransferred onto nitrocellulose membranes. Subsequently, the membranes were blocked with 5% skim (non-fat) milk in Tris-buffered saline containing 0.1% Tween-20 (TBST) for 1 h at room temperature before probing with the primary antibodies (anti-HcESP antibodies or normal rat IgG, 1:500 in TBST) overnight at 4 °C. After five washes, the membranes were incubated with horseradish peroxidase (HRP)-conjugated rabbit anti-goat IgG (H + L) secondary antibody (Sigma) in TBST (1:2000) for 1 h at 37 °C. Immunoreactions were detected using 3,3′-diaminobenzidine (DAB, Sigma) as a chromogenic substrate after 3-5 min of colour development.

### Immunofluorescence assay

Immunocytochemistry assays were performed to verify the interaction of HcESPs with goat T cells as previously described [11]. Briefly, freshly sorted T cells (1 × 10^6^ cells/mL) were incubated with 20 µg/mL HcESPs at 37 °C for 6 h. After five washes, the cells were fixed with 4% paraformaldehyde for 20 min at room temperature, permeabilized by 0.5% Triton X-100 in PBS for 5 min and blocked with 4% BSA in PBS for 30 min to minimize background staining. Subsequently, the cells were treated with rat anti-HcESP polyclonal antibody (1:100) or normal rat IgG (control) in a humidified atmosphere at 37 °C for 1 h, followed by staining with Cy3-coupled secondary antibody (1:500) at room temperature for 30 min. 2-(4-Amidinophenyl)-6-indole carbamidinedihydrochloride (DAPI, Sigma) was used for nuclear staining. Immunofluorescence-labelled cells were imaged at 100× magnification using a Zeiss LSM710 laser scanning confocal microscope (Zeiss, Jena, Germany). Digital images were analysed by Zen 2012 imaging software (Zeiss). Each experiment was performed in triplicate.

### Co-immunoprecipitation (co-IP) assays

Co-IP assays were performed to determine the proteins of HcESPs that interacted with goat T cells using Protein A/G PLUS-Agarose (Santa Cruz Biotechnology, Texas, USA) as described elsewhere [21]. The goat T cells were incubated with 20 µg/mL HcESPs at 37 °C with 5% CO_2_ for 6 h. After three washes with PBS, the cell pellets were lysed with Pierce™ IP Lysis Buffer (Thermo Fisher Inc, Rockford, IL, USA) containing Halt protease inhibitor cocktail and phosphatase inhibitor cocktail (Thermo Fisher). The cell lysates were collected after centrifugation at 12 000× rpm for 10 min at 4 °C, followed by preclearing with 2.0 μg of normal rat IgG and 40 μL of Protein A/G PLUS-Agarose at 4 °C for 30 min. After pretreatment, duplicate cell lysates (1 mg) were incubated overnight at 4 °C with rat anti-HcESP IgG as the IP sample and normal rat IgG as the negative control sample. Immune complexes were precipitated with 40 μL of Protein A/G PLUS-Agarose at 4 °C for 4 h. After four washes with IP lysis buffer, the immunoprecipitates were collected and resuspended in 80 μL of 1 × SDS loading buffer. The resulting protein samples were resolved on a 12% SDS-PAGE gel and then transferred onto nitrocellulose membranes. The membranes were probed with rat anti-HcESP IgG as the primary antibody for Western blot analysis. Each experiment was performed in triplicate.

### Liquid chromatography–tandem mass spectrometry (LC–MS/MS) and bioinformatics analysis

Identification of the immunoprecipitates was performed by in-solution trypsin digestion and LC–MS/MS analysis using a Q Exactive instrument (Thermo Finnigan, San Jose, CA, USA) at Shanghai Applied Protein Technology Co., Ltd., as previously described [18]. Based on the corresponding UniProt database of the *H. contortus* genome, the raw files from the LC–MS/MS tests were analysed using Mascot 2.2 search software (v.2.2, Matrix Science, London, UK), specifying carbamidomethyl (C) as the fixed modification and oxidation (M) as the dynamic modification and allowing less than two missed cleavages. Meanwhile, all the identified peptides were screened by false discovery rate (FDR) ≤ 0.01 and Mascot score ≥ 20. After the multi-analysis stated above, proteins with ≥ 2 unique peptides in three biological replicates were thought to be the identified interacting proteins. Gene Ontology (GO) annotation of parasite and host interacting proteins was performed for functional classification based on the categories of molecular function, cellular component and biological process using Blast2GO based on the BLASTP results.

### Cell viability

The effects of the HcESPs on the viability of goat T cells were determined using the cell counting kit-8 (CCK-8) assay (Dojindo, Kumamoto, Japan) as described elsewhere [11]. T cells activated with concanavalin A (ConA, 5 μg/mL, Sigma) were treated with serial dilutions of HcESPs (0, 10, 20, 40 and 80 µg/mL) at 37 °C with 5% CO_2_ in a humidified atmosphere. Following 24 h of incubation, 10 μL of CCK-8 reagent was added for another 4 h of incubation in the dark. The optical density at 450 nm (OD450) was measured using a microplate reader (Bio-Rad, Hercules, California, USA). Three independent experiments were performed, and each experiment was performed in triplicate.

### Cell apoptosis assay

Flow cytometry analysis of apoptosis was performed as previously described using the Annexin V-PE Kit (BD Biosciences, San Jose, California, USA) [21]. Briefly, freshly isolated T cells were incubated with different concentrations of HcESPs (0, 10, 20, 40 and 80 µg/mL) for 24 h followed by staining with Annexin V and 7-aminoactinomycin D (7-AAD) according to the manufacturer’s protocols. Unstimulated goat T cells were used as a negative control. The apoptosis rate was calculated from the percentage of early (AnnexinV^+^7AAD^−^) and late (AnnexinV^+^7AAD^+^) apoptotic T cells. Three individual experiments, each consisting of three replicates, were carried out.

### Cell proliferation assay

Cell proliferation assays were performed by directly measuring DNA synthesis using an Alexa Fluor 647 click-iT plus EdU flow cytometry kit (Thermo Fisher) according to the manufacturer’s instructions. 5-Ethynyl-2′-deoxyuridine (EdU, 10 μM) was added to the culture medium for a 12-h incubation in a humidified atmosphere at 37 °C with 5% CO_2_ at the end of the 12-h coincubation (0, 10, 20, 40, 80 µg/mL HcESPs with T cells) period. Subsequently, T cells were fixed with 4% paraformaldehyde in PBS and permeabilized with Click-iT saponin-based permeabilization and wash reagent, followed by Click-iT reaction to couple EdU with the Alexa Fluor 647 dye. After two washes with 3 mL of 1% BSA in PBS, T cells were treated with 7-AAD Staining Solution (BD Biosciences), and standard flow cytometry methods were used to determine the percentage of S-phase cells in the population. Each experiment consisting of three replicates was performed in triplicate.

### Cell cycle analysis

Cell cycle analysis was performed following the manufacturer’s DNA staining protocol for flow cytometry. During the 24-h coincubation with HcESPs (20 µg/mL) at 37 °C in a humidified atmosphere with 5% CO_2_, T cells were collected at different time points (0, 6, 12, 18 and 24 h), washed and fixed with ice-cold 75% ethanol. After incubation at −20 °C for 12 h, the cells were washed twice to remove the ethanol and resuspended in PI/RNase staining buffer (1 × 10^6^ cells/500 µL, BD Biosciences) for flow cytometry analysis. Three independent experiments were performed, and each experiment was performed in triplicate.

### Transcription analysis of candidate genes

T cells treated with various concentrations (0, 10, 20, 40 and 80 µg/mL) of HcESPs for 24 h were harvested for cell apoptosis analysis, and T cells treated with 20 µg/mL HcESPs for different durations (0, 6, 12, 18 and 24 h) were collected for cell cycle analysis. Total RNA samples were extracted, and the resulting cDNA was synthesized by reverse transcription PCR in accordance with the manufacturer’s specifications. Transcriptional analysis of candidate genes was conducted by real-time PCR with a standard procedure on a QuantStudio 3 Real Time PCR System (Applied Biosystems, Carlsbad, CA, USA) using published specific primers for endogenous reference genes and target genes (Additional file 1) [21, 23–30]. The amplification efficiencies and correlation coefficients were verified to be stable and similar, and the relative mRNA expression levels of candidate genes were calculated by the 2^−ΔΔCt^ method. Each experiment was performed in triplicate.

### Enzyme-linked immunosorbent assay (ELISA) for cytokine secretion

Detection of cytokine secretion was performed using goat IL-2, IL-4, IL-10, IL-17A, interferon (IFN)-γ and transforming growth factor (TGF)-β1 ELISA kits (Mlbio, Shanghai, China). Fresh isolated T cells activated by ConA were incubated with the presence of HcESPs (0, 10, 20, 40 and 80 µg/mL) for 24 h in a humidified atmosphere with 5% CO_2_ at 37 °C. The supernatants were collected and assayed for cytokine production according to the manufacturer’s protocols. The limits of detection were between 2 and 800 pg/mL depending on the analytic assay. Each experiment was performed in triplicate.

### Statistical analysis

Statistical analysis by one-way ANOVA for significant differences was performed using GraphPad Premier 6.0 software (GraphPad Prism, San Diego, CA, USA). *P* < 0.05 was considered to be statistically significant. Data are expressed as the mean ± standard deviation (SD).

## Results

### HcESP collection and rat anti-HcESP IgG generation

**Figure 1:**
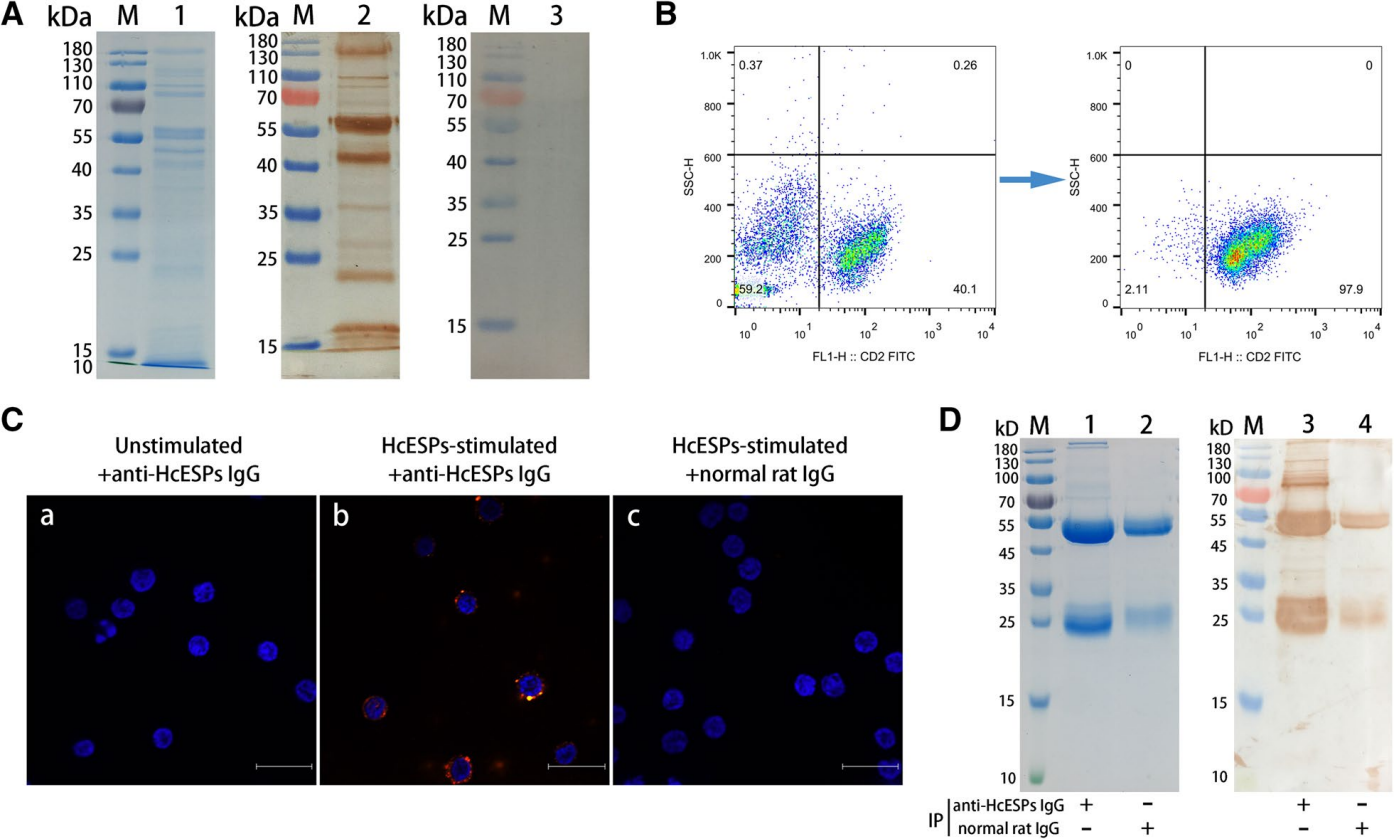
**Confirmation of the interaction between HcESPs and goat T cells and identification of the immune complexes from the interacting proteins by co-IP assays. A** Collection of HcESPs and generation of rat anti-HcESP IgG. Lane 1: SDS-PAGE analysis of HcESPs; lane 2: Immunoblot analysis of HcESPs using rat anti-HcESP IgG as the primary antibody; lane 3: Immunoblot analysis of HcESPs using normal rat IgG as the negative control. **B** Goat T cell sorting by MACS. The purity of the isolated T cells was above 95%, as indicated by flow cytometry analysis. **C** Binding of HcESPs to goat T cells in vitro. The immunocytochemistry assays were performed using rat anti-HcESP IgG or normal rat IgG (control). DAPI (blue) and Cy3-conjugated secondary antibodies (red) were utilized for dual staining. T cells stimulated in the absence (a) or presence (b) of HcESPs were incubated with rat anti-HcESP IgG as the primary antibody. T cells pretreated with HcESPs were incubated with normal rat IgG as the primary antibody (c). Scale bars, 50 µm. **D** The interaction between HcESPs and T cells was tested by co-IP assays. Lane 1: SDS-PAGE analysis of the cell lysates precipitated by rat anti-HcESP IgG; lane 2: SDS-PAGE analysis of the cell lysates precipitated by normal rat IgG (control); lane 3: Western blot analysis of the cell lysates precipitated by rat anti-HcESP IgG; lane 4: Western blot analysis of the cell lysates precipitated by normal rat IgG (control); lane M: standard protein molecular marker.

To identify the highly concentrated HcESPs obtained via in vitro culture, ~50 µg of HcESPs were separated by 12% SDS-PAGE gels. The Comus bright blue stain showed that the molecular weights of the collected HcESPs ranged from ~10 kD to ~180 kD (Figure 1A, lane 1). Western blot analysis revealed that rat anti-HcESP IgG (Figure 1A, lane 2) recognized all the bands of natural HcESPs distributed from ~10 kD to ~180 kD, but the control normal rat IgG (Figure 1A, lane 3) did not recognize any band, indicating that the rat anti-HcESP IgG was specific to the HcESPs.

### T cell sorting and the binding of HcESPs to goat T cells in vitro

Freshly sorted goat T cells with a purity of > 95% were obtained by positive selection and used for functional and immunological studies (Figure 1B). The interactions of HcESPs with goat T cells in vitro were investigated by immunocytochemistry assays. The immunostaining results showed that intense red fluorescence resulting from tagging HcESP proteins with specific rat anti-HcESP IgG was detected in HcESP-pretreated T cells (Figure 1C, panel b), indicating the cytomembrane and cytoplasmic localization of HcESPs. However, no red fluorescence was observed in either unstimulated cells labelled with rat anti-HcESP IgG (Figure 1C, panel a) or HcESP-pretreated cells labelled with normal rat IgG (Figure 1C, panel c).

### Co-IP assays and LC–MS/MS analysis

Based on the positive results of the immunostaining assay, co-IP assays were conducted in HcESP-stimulated T cells. The immune complexes resolved on 12% SDS-PAGE gels were extracted from the interacting proteins using rat anti-HcESP IgG (Figure 1D, lane 1) or normal rat IgG (Figure 1D, lane 2) as the IP antibody. Consistent with the observation from Coomassie brilliant blue staining, Western blot analysis demonstrated that the interacting proteins were present in rat anti-HcESP IgG-precipitated immune complexes, as indicated by the multiple detected bands ranging from ~20 kD to ~180 kD (Figure 1D, lane 3). However, no significant protein bands, except those for the heavy and light chains of normal rat IgG, were detected in normal rat IgG-precipitated immune complexes (Figure 1D, lane 4). The immunoprecipitates detected by LC–MS/MS analysis were identified by extraction and quantification of the peptides obtained by digestion. A total of 114 matched ES proteins with unique peptides and 15 T cell binding partners are given in Additional file 2 and Additional file 3, respectively. Among the interacting ES proteins, a variety of enzymes, namely, hydrolase, proteases, acyltransferase, kinases, phosphatases, and lipases, were identified. Meanwhile, protein transporters, ion-binding proteins, conserved regulatory proteins such as ubiquitin and Ras, and conserved structural proteins such as histones, actins, collagen and actin-binding proteins were identified. Furthermore, proteins with unknown functions, as well as novel proteins that are yet to be annotated, were also detected in this study (Additional file 2). Importantly, the goat FasL protein was confirmed as being present among the 15 T cell binding proteins (Additional file 3). The association of FasL with its receptor Fas may trigger the transmembrane signalling of an apoptotic pathway that plays a vital role in cellular development and immune regulation [31].

### Bioinformatics analysis

**Figure 2:**
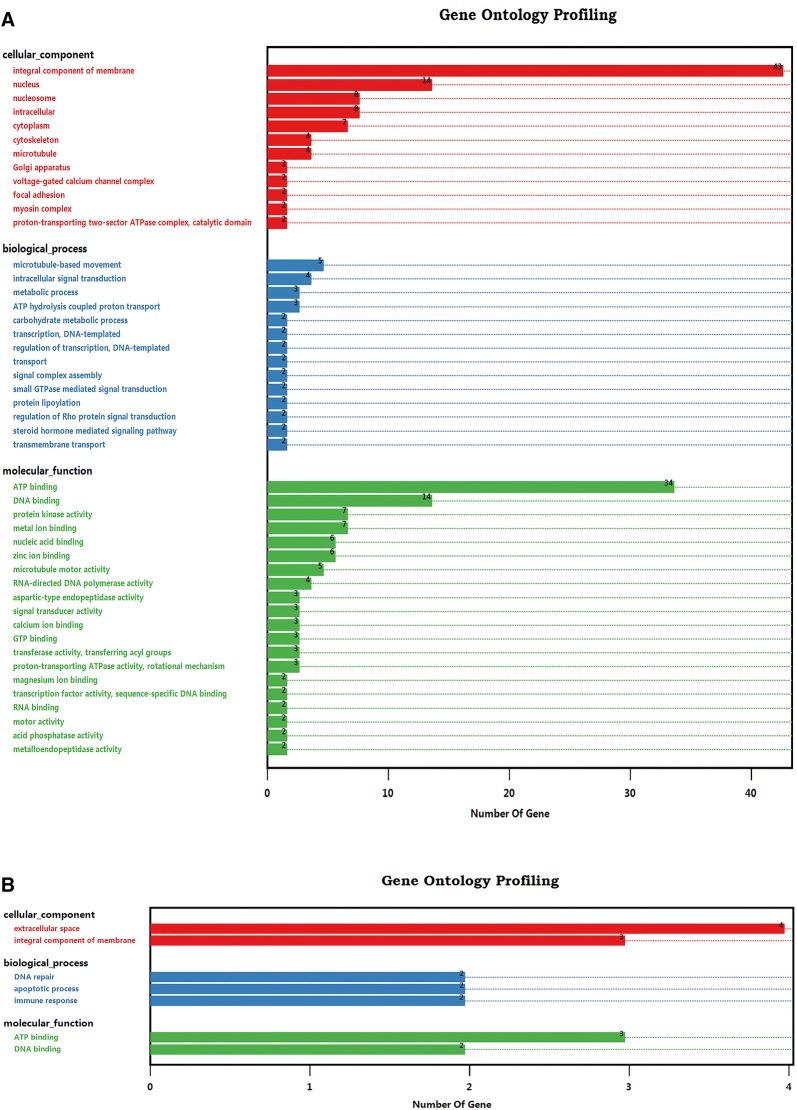
**GO annotation of the interacting ES proteins and T cell receptors based on the categories of cellular component, biological process and molecular function. A** Gene Ontology categories of the identified ES proteins binding to goat T cells. **B** Gene Ontology categories of T cell binding receptors.

Blast2GO was used to identify the GO terms for the 114 ES proteins and 15 T cell binding receptors. Of these 114 ES proteins, 98 were annotated into 12 subcategories of cellular component terms. In contrast, 35 proteins were enriched in 14 subcategories of biological process terms, and 62 proteins were assigned to 20 subcategories of molecular function terms (Figure 2A). Consistent with previous proteomic analyses [18, 32, 33], serine-threonine/tyrosine protein kinases and histidine/tyrosine phosphatases were enriched in protein kinase/phosphatase activity subcategories in this study. Both protein kinases and phosphatases are key regulators of cellular functions and are particularly prominent in signal transduction and coordination involved in the cell cycle, cell survival, protein–protein interaction, and inflammation [34, 35]. Notably, 67.5% of the identified ES proteins (*n* = 77) were enriched in binding activity terms, namely, ion binding, substrate compound binding, and nucleic acid binding. Proteins involved in these functions are normally associated with energy metabolism, signalling, and transcription [36–38]. As external stimuli, these exogenous proteins may bind to host T cells, interfere with the intracellular homoeostatic balance and disrupt multitudinous cellular functions. Additionally, among the GO terms of T cell binding proteins, cellular component terms were allocated to the extracellular space (*n* = 4) and integral component of membrane (*n* = 3) subcategories. Biological process terms were allocated to the DNA repair (*n* = 2), apoptotic process (*n* = 2) and immune response (*n* = 2) subcategories, and molecular function terms were grouped into the DNA binding (*n* = 3) and ATP binding (*n* = 2) subcategories (Figure 2B). In particular, as GO annotation revealed that T cell binding receptors were enriched in biological process terms associated with cell apoptosis and immunomodulation, which were mainly attributed to the functional annotation of FasL, it is likely that the external stimuli of HcESPs played a pivotal role in the regulation of T cell survival and growth, as well as the T cell immune response.

### HcESPs affected cell viability and induced cell apoptosis

**Figure 3:**
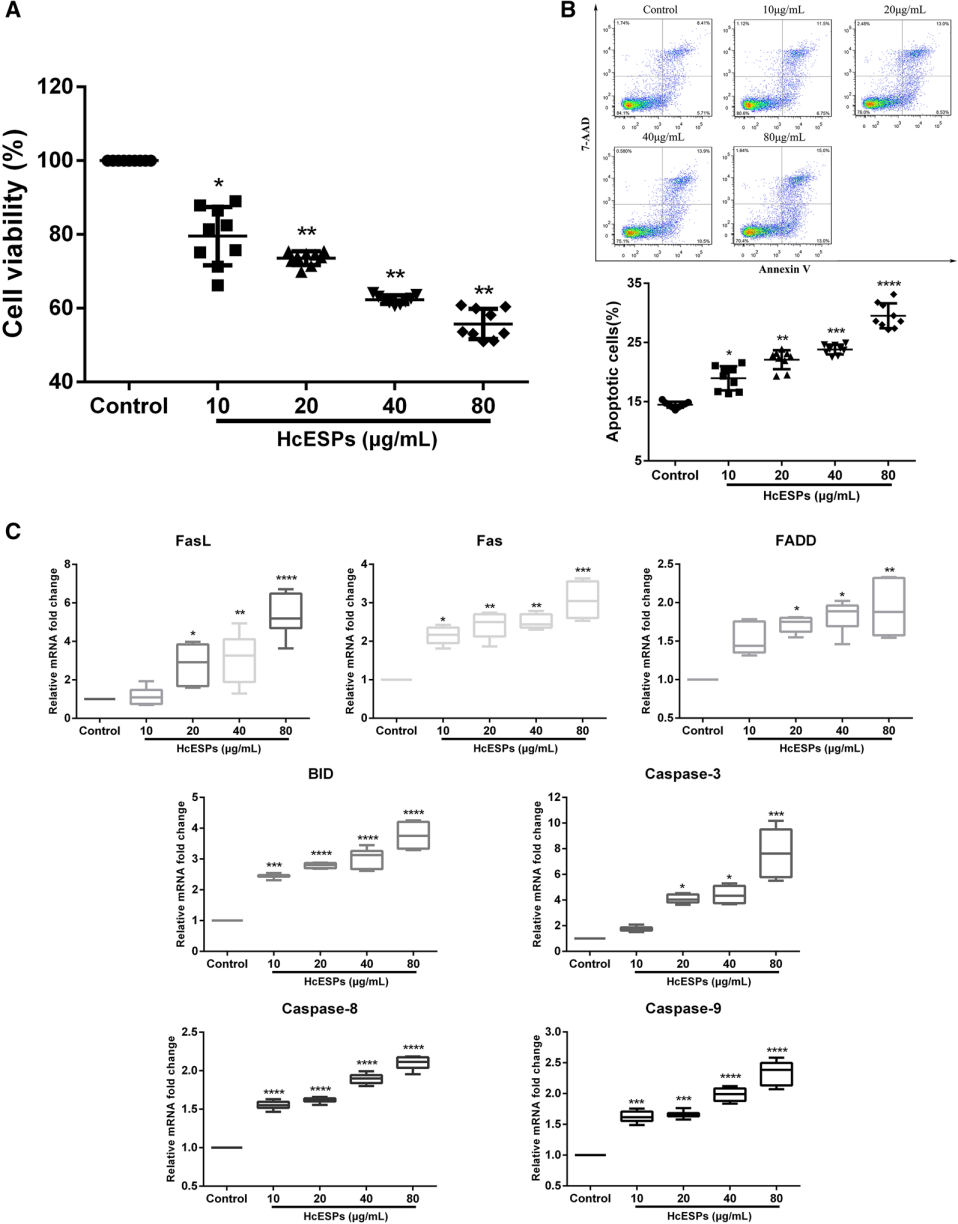
**HcESPs significantly affected cell viability and promoted T cell apoptosis. A** Treatment with HcESPs significantly inhibited T cell viability. Cell viability tests were performed by CCK-8 incorporation, and the cell viability index was determined by setting the OD450 values of the control group as 100%. **B** Flow cytometry analysis of T cell apoptosis in response to HcESPs. Apoptosis of T cells was determined by staining with Annexin V-PE and 7-AAD. The apoptosis rate was calculated from the percentage of early (AnnexinV^+^7AAD^−^) and late (AnnexinV^+^7AAD^+^) apoptotic T cells. **C** The mRNA transcripts of candidate genes in T cells stimulated with HcESPs. The results presented here are representative of three independent experiments. The data are presented as the mean ± SD, minimum to maximum; *P < 0.05, **P < 0.01, ***P < 0.001, ****P < 0.0001 vs the control group.

To verify the new clues from GO annotation indicating that HcESP stimuli might affect T cell growth and survival, we further investigated the biological effects of HcESPs on the viability of goat T cells. Consistent with this hypothesis, our data showed that the viability of goat T cells was significantly inhibited by treatments with 10 μg/mL (*P* < 0.05), 20 μg/mL (*P* < 0.01), 40 μg/mL (*P* < 0.01) and 80 μg/mL (*P* < 0.01) HcESPs (Figure 3A). Furthermore, an Annexin V-PE/7-AAD dual staining kit was used to test the pro-apoptotic effects of HcESPs on T cells in this study. Flow cytometry analysis demonstrated that treatment with HcESPs dramatically promoted T cell apoptosis in a dose-dependent manner compared to the control group (*P* < 0.05) (Figure 3B). Concurrently, transcriptional analysis of key genes in Fas-mediated apoptotic signal pathways further confirmed the induction of T cell apoptosis by HcESPs. Treatment with ≥ 20 μg/mL HcESPs significantly upregulated the mRNA transcripts of FasL (*P* < 0.05), Fas (*P* < 0.01), FADD (*P* < 0.05), BID (*P* < 0.0001), Caspase 3 (*P* < 0.05), Caspase 8 (*P* < 0.0001) and Caspase 9 (*P* < 0.001) (Figure 3C). Taken together, these data revealed that HcESP stimuli induced T cell apoptosis via the upregulation of the transcription of several essential genes in the apoptosis pathway.

### HcESPs suppressed cell proliferation and induced cell cycle arrest

**Figure 4:**
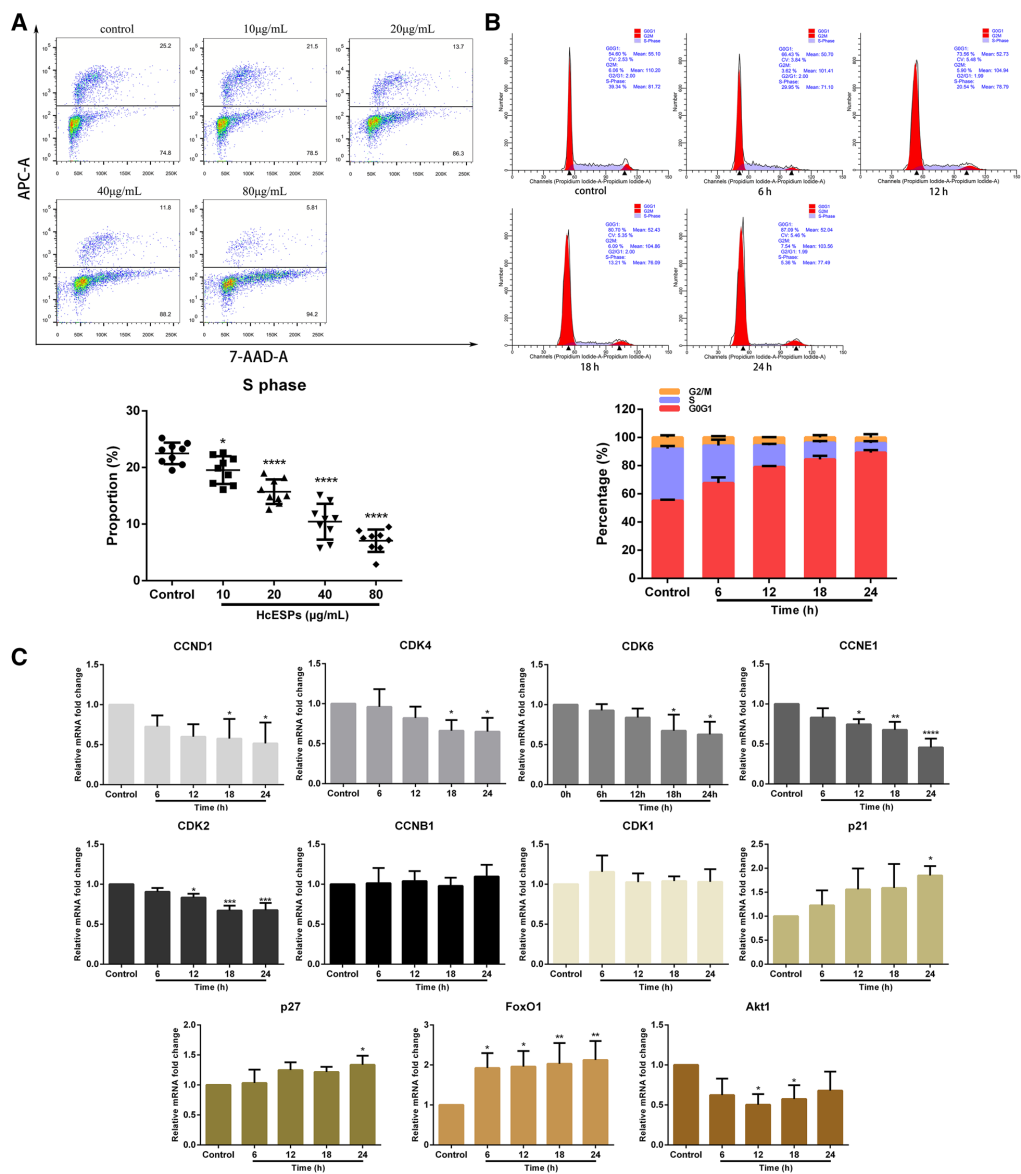
**HcESPs suppressed cell proliferation and induced T cell cycle arrest at G1 phase. A** Flow cytometry analysis of T cell proliferation in response to HcESP treatment. T cell proliferation was determined by staining with 7-AAD and EdU-APC. The proliferation rate was calculated from the percentage of T cells in S phase. **B** Cell cycle analysis of T cells was performed by flow cytometry using PI/RNase staining reagent. HcESPs caused cell cycle arrest at the G1 phase. **C** Relative levels of CCND1, CCNE1, CCNB1, CDK4, CDK6, CDK2, CDK1, p21, p27, FoxO1 and Akt1 transcription in goat T cells treated with HcESPs. The results presented here are representative of three independent experiments. The data are presented as the mean ± SD; *P < 0.05, **P < 0.01, ***P < 0.001, ****P < 0.0001 vs the control group.

As apoptosis, proliferation and the cell cycle are interconnected cellular movements, we next explored the influence of HcESP stimuli on the cell proliferation and cell cycle of host T cells. In a dose-dependent manner, treatment with HcESPs significantly inhibited the proliferation of T cells in vitro, as indicated by the decreasing percentage of cells in S phase compared with control cells (*P* < 0.05) (Figure 4A). Given that the treatment with 20 μg/mL HcESPs had significant biological effects on cell viability, apoptosis and proliferation, as well as the transcription of certain key genes, we next treated T cells with 20 µg/mL HcESPs for cell cycle determination. Flow cytometry analysis with PI staining revealed that HcESP stimuli induced cell cycle arrest at G1 phase in a time-dependent manner, as indicated by the increasing percentage of cells in G1 phase (*P* < 0.01) and the decreasing percentage of cells in S phase (*P* < 0.01) (Figure 4B). Consistent with the above findings, real-time PCR analysis of key genes in G1/S checkpoints and G2/M checkpoints showed that the transcription of CCND1 (*P* < 0.05), CCNE1 (*P* < 0.05), CDK4 (*P* < 0.05), CDK6 (*P* < 0.05) and CDK2 (*P* < 0.05) was significantly downregulated, whereas no significant transcriptional changes in CCNB1 or CDK1 were observed (Figure 4C). In addition, the transcription of essential genes in the Akt/PKB pathway that regulate cell proliferation and cell growth was also detected in this study. The transcripts of FoxO1 (*P* < 0.05), p21 (*P* < 0.05) and p27 (*P* < 0.05) were dramatically enhanced, while Akt1 transcription was significantly suppressed (*P* < 0.05) (Figure 4C). Collectively, HcESP stimuli restrained T cell proliferation and caused T cell cycle stalling at the G1 phase.

### ELISA assays

**Figure 5:**
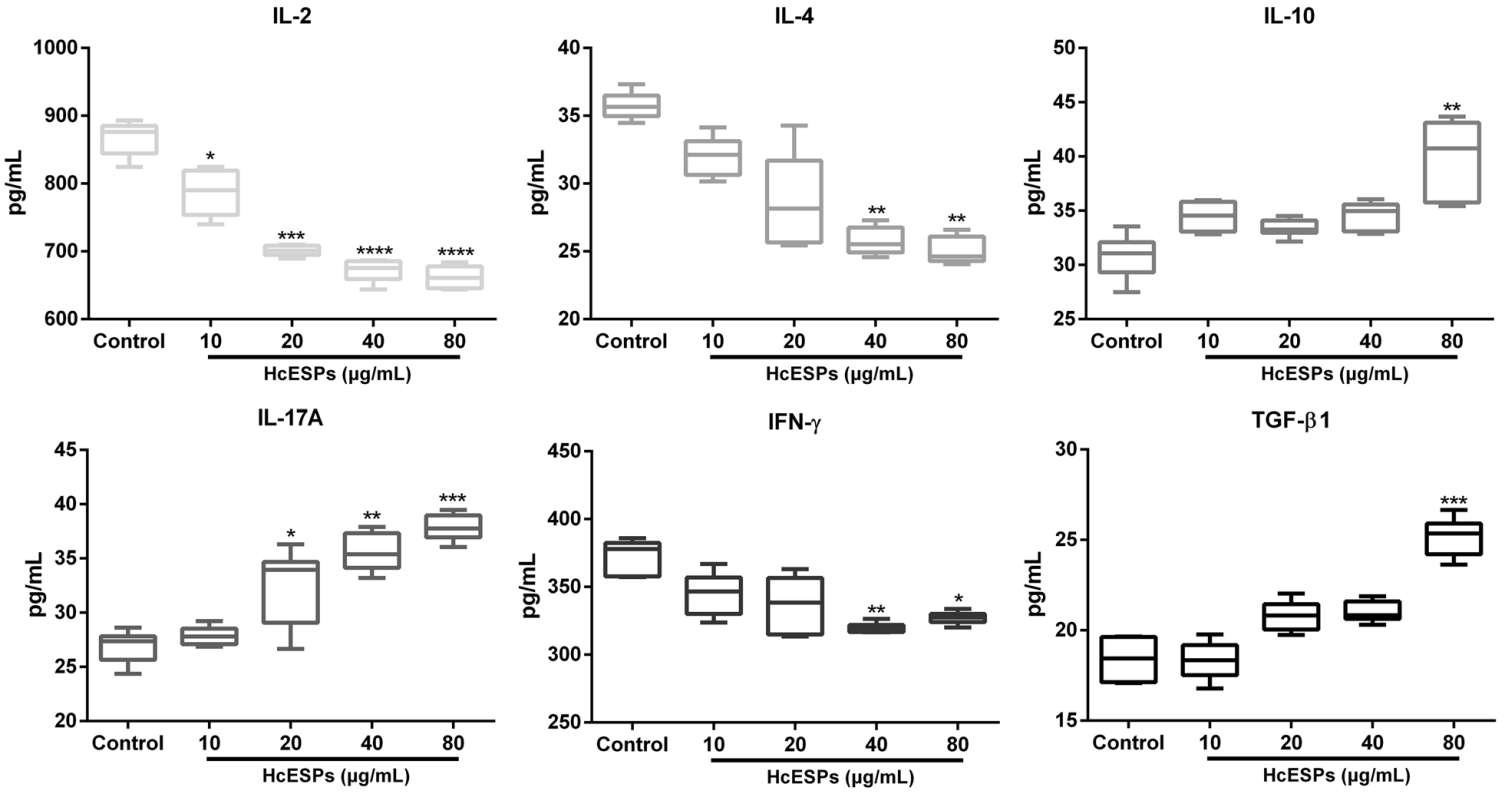
**HcESP stimuli changed the cytokine secretion profiles of T cells in vitro.** Goat T cells were incubated in the presence or absence of HcESPs for 24 h. The secretion of IL-2, IL-4, IL-10, IL-17A, IFN-γ and TGF-β1 in the culture supernatant was detected by ELISA. The results presented here are representative of three independent experiments. The data are presented as minimum to maximum; *P < 0.05, **P < 0.01, ***P < 0.001, ****P < 0.0001 vs the control group.

To investigate the modulatory effects of HcESPs on T cell cytokine production, the secretion of IL-2, IL-4, IL-10, IL-17A, IFN-γ, and TGF-β1 was examined by ELISA in this study. The results showed that goat T cells exposed to HcESPs changed their cytokine production profile (Figure 5). The production of IL-2 was predominantly inhibited by stimulation with 10 μg/mL (*P* < 0.05), 20 μg/mL (*P* < 0.001), 40 μg/mL (*P* < 0.0001) and 80 μg/mL (*P* < 0.0001) HcESPs. IL-17A secretion was significantly promoted by treatment with 20 μg/mL (*P* < 0.05), 40 μg/mL (*P* < 0.01) and 80 μg/mL (*P* < 0.001) HcESPs. Meanwhile, treatment with 40 μg/mL and 80 μg/mL HcESPs dramatically inhibited IL-4 (*P* < 0.01 and *P* < 0.01, respectively) and IFN-γ production (*P* < 0.01 and *P* < 0.05, respectively). Importantly, a high dose of HcESPs (80 μg/mL) promoted the secretion of IL-10 (*P* < 0.01) and TGF-β1 (*P* < 0.001) (Figure 5). Taken together, the results show that HcESP stimuli mainly played an immunosuppressive role in the T cell immune response via alteration of cytokine secretion profiles.

## Discussion

For decades, significant efforts have been made to identify the composition and structure of HcESPs from different developmental stages to better understand the pathophysiology and develop novel controls. Yatsuda et al. identified 107 adult HcESPs recognized by anti-*H. contortus* serum for the first time [39], whereas Wang et al. recently characterized 878 unique proteins in ES products from different life-cycle stages [40]. Here, we identified 114 adult ES proteins that interacted with goat T cells through co-IP and LC–MS/MS analysis. Notably, a cascade of proteolytic peptidases, including aspartic peptidases and metallopeptidases, was identified from these interacting ES proteins and is postulated to play crucial roles in larval development, adult survival and reproduction via the digestion or degradation of host haemoglobin [41, 42]. Similarly, comparable expression of these peptidase family members in ES products was reported in a series of blood-feeding nematodes, such as *A. caninum* [43], *Necator americanus* [44], and *Ancylostoma ceylanicum* [45]. Simultaneously, other essential molecules, e.g., C-type lectins (CLECs) and venom allergen-like proteins (VALs), were detected in the current study. As members of the lectin superfamily, CLECs engage in a diverse set of immune regulation processes by glycan binding and are as indispensable to nematode survival as the secretome [46, 47]. Given their wide distribution and potential binding with the host, CLECs have been speculated to be immunomodulators in parasite-host interactions or parasitic immune evasion [47]. In addition, recent studies reported abundantly secreted VALs in ES products that caused severe damage to host tissue, favouring the establishment of persistent infections during parasitism, and VALs could inhibit innate immune responses and remodel the extracellular matrix [48].

In this study, our preliminary bioinformatics analysis revealed that HcESP stimuli might affect T cell growth and survival, based on the identification of FasL, a binding ligand of Fas. Apoptosis can be induced through the activation of death receptors, including Fas, TNFαR, DR3, DR4, and DR5, by their respective ligands [49]. Consistent with the GO annotation, our data proved that HcESP stimuli dramatically inhibited T cell viability and induced cell apoptosis. Generally, death receptor ligands initiate signalling via receptor oligomerization, which in turn results in the recruitment of specialized adaptor proteins and activation of caspase cascades [31]. Regarding Fas-mediated signalling, Fas trimerization resulting from binding to FasL leads to recruitment of the initiator Caspase-8 via the adaptor protein FADD, inducing Caspase-8 oligomerization and activation via autocatalysis. Subsequently, activated Caspase-8 stimulates apoptosis by directly cleaving and activating Caspase-3 or alternatively cleaving Bid, which is a pro-apoptotic Bcl-2 family protein. Then, truncated Bid (tBid) translocates to mitochondria, facilitating the release of cytochrome c to activate Caspase-9 and Caspase-3 sequentially [50, 51]. In this study, HcESPs exerted modulatory effects on the transcript levels of several essential genes in the Fas-mediated death receptor pathway. The mRNA transcription of FasL, Fas, FADD, Bid, Caspase-3, Caspase-8 and Caspase-9 was notably upregulated due to HcESP stimulation, indicating a potential mechanism of HcESP-induced intrinsic and extrinsic apoptosis of T cells.

Cell apoptosis, the cell cycle, and cell proliferation are fundamental and ultimately linked processes. Analogous to its pro-apoptotic effects, HcESP stimuli repressed cell proliferation in a dose-dependent manner. In eukaryotic cells, cell cycle progression is controlled by the G1/S checkpoint via CDK4/6-Cyclin D and CDK2-Cyclin E kinase complexes and the G2/M checkpoint via the Cyclin B-cdc2 (CDK1) complex [52, 53]. Here, PI/RNase staining results revealed that HcESP stimuli induced cell cycle arrest at the G1 phase in a time-dependent manner. Furthermore, the commitment of T cells to enter from the G1/S phase into G2/M phase was prevented by HcESPs through the downregulation of CCND1, CDK4/6, CCNE1, and CDK2 transcription. The serine/threonine kinase Akt contributes to cell proliferation by phosphorylating the CDK inhibitors p21 and p27, both of which have direct inhibitory modification effects on CDK2 [54]. Akt also has multistep inhibitory modifying effects on Cyclin D via GSK-3β [55]. Importantly, Akt is a major mediator of cell survival through direct inhibition of pro-apoptotic proteins such as Bad or inhibition of pro-apoptotic signals generated by transcription factors such as FoxO [56]. Based on these associations, we found that Akt1 transcription was significantly suppressed, whereas p21, p27 and FoxO1 transcription was strongly promoted, in HcESP-treated T cells. Given that FoxO1 induces apoptosis by upregulating the pro-apoptotic molecule FasL and CDK2 directly phosphorylates FoxO1 [57], the associations among FasL, FoxO1, CDKs, Cyclin proteins and Akt represent complex regulation by HcESP stimuli that can act via the alteration of mRNA transcription of key genes in the AKT/PKB pathway to mediate cell cycle arrest and apoptosis. Collectively, this could be the mechanism by which HcESPs regulate cell apoptosis, the cell cycle and the proliferation of goat T cells. However, due to the lack of available goat immune reagents, we only checked the expression of several key molecules at the transcription level in this study. Details regarding the regulation of these molecules at the protein level, along with the associated pathways, merit further investigation. As there is currently no literature revealing the physiological dose range of HcESPs, we employed serial dilutions of HcESPs (0, 10, 20, 40 and 80 µg/mL) for functional studies and demonstrated that 20 µg/mL was the best reference concentration that had a significant impact on cell viability, apoptosis, proliferation and the cell cycle. However, under the circumstance of natural infection, whether the accumulation of HcESPs in *H. contortus*-infected goats could reach this relevant dose (20 µg/mL) to induce notable suppression effects on T cells is still unclear, and further efforts are needed to validate this hypothesis.

Generally, immunomodulation by ES proteins of parasitic helminths has several predominant features: mediating Th2 responses as exemplified by the secretion of IL-4; inducing the generation of anti-inflammatory cytokines, including IL-10 and TGF-β; inhibiting lymphocyte activation; blocking pro-inflammatory and Th1 cytokines such as IL-2 and IFN-γ; and regulating Treg and Th17 responses [4, 58]. Consistent with these findings, HcESPs significantly suppressed the secretion of IL-2, IL-4 and IFN-γ, indicating that HcESP stimuli exerted critical control effects on Th2 and Th1 responses. Although the reduced IL-2, IL-4 and IFN-γ secretion might be associated with the inhibition of cell viability and proliferation, we could not precisely determine IL-2/IL-4/IFN-γ production on a per cell basis here, and we could not determine the exact proportion of IL-2^+^/IL-4^+^/IFN-γ^+^ T cells among total T cells. Thus, whether the reduction in IL-2/IL-4/IFN-γ production resulted from HcESP stimuli causing reduced numbers of live IL-2^+^/IL-4^+^/IFN-γ^+^ T cells, reduced numbers of differentiated IL-2^+^/IL-4^+^/IFN-γ^+^ T cells, or increased numbers of hyporesponsive IL-2^+^/IL-4^+^/IFN-γ^+^ T cells remains unclear, and further efforts are needed to address this issue. In a previous study, a Galectin-9 homologue derived from adult *Toxascaris leonina* restrained inflammatory reactions by inhibiting Th1 and Th2 cytokine production by enhancing TGF-β and IL-10 production [59]. Similarly, a high dose of HcESPs also upregulated IL-10 and TGF-β1 secretion to inhibit the host inflammatory response. Additionally, TGF-β induces apoptosis via death-associated protein 6 (DAXX), another binding receptor of Fas [60]. Therefore, increased TGF-β1 secretion may exacerbate Fas-mediated apoptosis via a separate apoptotic pathway. Recent studies demonstrated the inhibition of Th17 differentiation and IL-17 secretion by *Echinococcus granulosus* and *Acanthocheilonema viteae* ES proteins [61, 62]. Instead, the secretion of the pro-inflammatory cytokine IL-17A was notably promoted by HcESP stimuli in this study, indicating that HcESPs may not induce apoptosis of the Th17 subset. In addition, TGF-β contributes to Th17 formation and IL-17 secretion [63, 64], and it is likely that increased TGF-β1 production may function as the key factor to facilitate IL-17A production.

As one of the most intensive research areas, a pleiotropic range of immunomodulatory activities of ES proteins has been determined in numerous species of GIhelminths, including *H. polygyrus* [65], *Teladorsagia circumcincta* [66], *A. caninum* [67], and *N. americanus* [68]. However, the role of individual ES components is still being elaborated or has yet to be determined in most instances. In our previous work, two novel ES proteins, namely, Y75B8A.8 and HcTTR, were validated as binding partners and novel antagonists of goat IL-2 and IL-4, respectively, and were shown to block downstream effectors [69, 70]. Although we employed proteomic approaches to identify a variety of potential immunomodulators, such as kinases, hydrolases, proteases, lipases, phosphatases and CLECs, in this study, the exact molecules that regulate/interact with T cell FasL directly/indirectly at the parasite-host interface, along with the cascade of cellular responses, still need to be further investigated.

## Supplementary information

**Additional file 1: Primer sequences for the transcription analysis of apoptosis and cell cycle.**

**Additional file 2: Full List of identified ES proteins interacting with goat T cells in vitro by Co-IP and LS–MS/MS.**

**Additional file 3: Full list of identified T cell binding receptors in vitro.**

## Data Availability

The datasets supporting the conclusions of this article are included within Additional file 1, Additional file 2 and Additional file 3.

## Abbreviations

ES: excretory-secretory
HcESPs: *Haemonchus contortus* ES proteins
LC–MS/MS: liquid chromatography-tandem mass spectrometry
Fas: TNF superfamily receptor 6
Akt: AKT8 virus oncogene cellular homologue
IL: interleukin
TGF: transforming growth factor
IFN: interferon
GI: gastrointestinal
PBMCs: peripheral blood mononuclear cells
FCS: foetal calf serum
co-IP: coimmunoprecipitation
GO: Gene ontology
FADD: Fas-associated protein with death domain
BID: a BH3 domain-only death agonist protein
Caspases: cysteine proteases with aspartate specificity
CDK: cyclin-dependent kinase
FoxO1: forkhead box protein O1
