# Supplementary material for: Proteomic analysis revealed T cell hyporesponsiveness induced by Haemonchus contortus excretory and secretory proteins
Source: Vet Res. 2020 May 13;51:65. doi: 10.1186/s13567-020-00790-0 (PMC7222441; doi:10.1186/s13567-020-00790-0)
Supplement: Supplementary file 1 — Additional file 1: Primer sequences for the transcription analysis of apoptosis and cell cycle. [file 13567_2020_790_MOESM1_ESM.docx]

**Additional file 1: Table S1. Primer sequences for the transcription analysis of apoptosis and cell cycle**.

| **Gene Name** | **Primer Sequence (5’-3’)** | **Reference** | **Size (bp)** |
| --- | --- | --- | --- |
| beta-actin | F: CACCACACCTTCTACAAC  R: TCTGGGTCATCTTCTCAC | [21] | 106 |
| GAPDH | F: CCTGGAGAAACCTGCCAAGT  R: GCCAAATTCATTGTCGTACCA | [23] | 214 |
| 18S rRNA | F: GTAACCCGTTGAACCCCATT  R: CCATCCAATCGGTAGTAGCG | [23] | 151 |
| FasL | F: AGCGGCTCATTTAACAGGCA  R: CCATGTCCTGGGGGTACCTA | [24] | 233 |
| Fas | F: GTCCTCCTGGCAAACGTAAA  R: CTCTTGTCTGTGTACTCGTTCC | NM_001314235.1 | 97 |
| FADD | F: TGCTTCCTAACTACAGGGTTTC  R: CGTAAGAGGTTTGCCTTCCA | XM_018043148.1 | 126 |
| BID | F: TGATCCAGGCTCAGAGAAGA  R: GTACGTTAATGCAGCAAGTCAAA | XM_018048713.1  XM_018048714.1 | 95 |
| Caspase 3 | F: CATTATTCAGGCCTGCCGAG  R: CTCGAGCTTGTGAGCGTACT | [25] | 220 |
| Caspase 8 | F: TTAGCATAGCACGGGAGCAG  R: GTCAGCTCATAGATGGGGGC | [25] | 280 |
| Caspase 9 | F: GGGAAATGCTGATCTGGCCT  R: CAGCCGTGAGAGAGGATGAC | [25] | 279 |
| CCND1 | F: GGTCCTGGTGAACAAACTC  R: TTGCGGATGATCTGCTT | [26] | 114 |
| CDK4 | F: CGTTGGCTGTATCTTTGC  R: GATTCGCTTGTGTGGGTT | [27] | 256 |
| CDK6 | F: AGAGTGATTGCAGCTTTATGTCCA  R: TGCCCAGGTTGCTCACTTC | [28] | 157 |
| CCNE1 | F: GGGACAAGCACCTTATGCAAC  R: GTGTTGCCATATACCGATCAAAGA | [28] | 153 |
| CDK2 | F: CTGCACCGAGACCTTAAACCTCA  R: GCTCGGTACCACAGAGTCACCA | [28] | 140 |
| CCNB1 | F: AGCGGATCCAAACCTTTGTAGTG  R: CAATGAGGATGGCTCTCATGTTTC | [28] | 137 |
| CDK1 | F: CCAATAATGAAGTGTGGCCAGAAG  R: AGAAATTCGTTTGGCAGGATCATAG | [28] | 164 |
| FoxO1 | F: CCCAGAGTCAGCACAATGAA  R: GATATGGGCTAGGTCAACAGAAG | XM_018056686.1 | 93 |
| p21 | F: CTAAGTGGGCAAATATGGGTCTGG  R: CAGGATGCTACAGGAGCTGGAAG | [29] | 107 |
| P27 | F: AAACCCAGAGGACACGCATT  R: GGCAGGTCGCTTCCTTATCC | [29] | 100 |
| Akt1 | F: GTACTCCTTCCAGACACACGACC  R: TACACCACGTTCTTCTCCGAG | [30] | 174 |
