# Supplementary material for: Proteomic analysis revealed T cell hyporesponsiveness induced by Haemonchus contortus excretory and secretory proteins
Source: Vet Res. 2020 May 13;51:65. doi: 10.1186/s13567-020-00790-0 (PMC7222441; doi:10.1186/s13567-020-00790-0)
Supplement: Supplementary file 2 — Additional file 2: Full List of identified ES proteins interacting with goat T cells in vitro by Co-IP and LS–MS/MS. [file 13567_2020_790_MOESM2_ESM.docx]

**Additional file 2: Table S2. Full List of identified ES proteins interacting with goat T cells in vitro by Co-IP and LS-MS/MS.**

| **Protein Description** | **Uniprot ID** | **Total peptide count** | **Unique**  **Pep Count** | **Cover Percent (%)** | **MW(Da)** | **PI** |
| --- | --- | --- | --- | --- | --- | --- |
| Histone H4 | U6PV53 | 18 | 4 | 29.13% | 11369.2 | 11.2 |
| Spectrin repeat and Dbl homology (DH) domain containing protein | U6P3X8 | 12 | 4 | 1.16% | 188809 | 6.05 |
| Histone core domain containing protein | U6PL99 | 9 | 4 | 7.41% | 21113.2 | 10.57 |
| Histone H3 | U6PZ56 | 9 | 3 | 10.29% | 15327.7 | 11.27 |
| Golgi autoantigen, golgin subfamily | U6PT55 | 9 | 3 | 0.86% | 231651 | 4.87 |
| Peptidase M12A domain containing protein | U6PF22 | 57 | 2 | 2.80% | 47452.6 | 9.25 |
| Arginyl tRNA synthetase | U6PXK8 | 12 | 2 | 1.39% | 80478.4 | 6.54 |
| Signal transducer and activator of transcription | U6PHB5 | 11 | 2 | 0.87% | 78333.1 | 6.13 |
| Mediator complex domain containing protein | U6NGV5 | 11 | 2 | 0.39% | 138367 | 8.94 |
| Peptidase A1 domain containing protein | U6Q069 | 11 | 2 | 1.40% | 46735.5 | 5.52 |
| Inner centromere protein domain containing protein | W6NE27 | 11 | 2 | 0.54% | 106347 | 9.27 |
| Major sperm protein | W6NAL3 | 11 | 2 | 3.57% | 13847.5 | 5.26 |
| alpha-1,2-Mannosidase | U6PP78 | 11 | 2 | 2.22% | 61239 | 6.09 |
| HAP1 domain containing protein | U6PHE1 | 11 | 2 | 0.87% | 63580.4 | 5.63 |
| Neurotransmitter-gated ion-channel ligand-binding protein | U6NTA9 | 11 | 2 | 0.69% | 83685.7 | 5.78 |
| Protein-tyrosine phosphatase domain containing protein | U6P6N1 | 11 | 2 | 0.64% | 87905.4 | 4.64 |
| Abhydrolase domain-containing protein | U6P5P3 | 10 | 2 | 2.56% | 34379.9 | 6.55 |
| Potassium channel and Ion transport 2 and Calmodulin-binding | U6NI12 | 10 | 2 | 0.78% | 72259.8 | 9.61 |
| Protein-tyrosine phosphatase domain containing protein | U6P2X2 | 10 | 2 | 0.61% | 92379.5 | 4.6 |
| Phospholipid glycerol acyltransferase domain containing protein | U6NIZ5 | 10 | 2 | 2.80% | 61340.7 | 5.88 |
| Peptidase M12A domain containing protein | U6P7J6 | 10 | 2 | 2.96% | 37196.6 | 8.35 |
| Na+ channel domain containing protein | U6NLC4 | 10 | 2 | 0.92% | 61081.7 | 8.23 |
| Ribosome biogenesis protein Nop16 domain containing protein | U6P321 | 10 | 2 | 0.84% | 68028.5 | 9.36 |
| Uncharacterized protein | U6NH29 | 10 | 2 | 3.11% | 17347.9 | 10.73 |
| WD40 repeat domain containing protein | U6P3X1 | 10 | 2 | 1.27% | 44491.4 | 5.46 |
| Ribosomal protein L24e domain containing protein | U6NVF6 | 10 | 2 | 2.53% | 22831.7 | 10.37 |
| Uncharacterized protein | U6PM36 | 10 | 2 | 3.76% | 14882 | 8.59 |
| Ankyrin and SOCS protein domain containing protein | U6PA90 | 10 | 2 | 1.05% | 53263.8 | 6.68 |
| NADH:ubiquinone oxidoreductase intermediate-associated protein | U6PZ35 | 10 | 2 | 1.45% | 40201.4 | 8.13 |
| Protein kinase domain containing protein | U6PE41 | 10 | 2 | 1.32% | 43901.8 | 9.03 |
| Uncharacterized protein | U6NT30 | 10 | 2 | 1.36% | 43055.5 | 4.89 |
| CK1/WORM6 protein kinase | W6NFI2 | 10 | 2 | 0.85% | 67517.1 | 9.59 |
| Tetratricopeptide repeat protein 26 isoform 1 | U6PP07 | 10 | 2 | 0.91% | 63565.4 | 6.06 |
| Adhesion regulating molecule domain containing protein | W6NB91 | 10 | 2 | 1.66% | 37947.5 | 5.29 |
| Uncharacterized protein | U6NVS8 | 10 | 2 | 6.02% | 9310.55 | 6.01 |
| Acetylcholine receptor alpha subunit (Fragment) | Q9NIU9 | 9 | 2 | 10.20% | 5429.6 | 11 |
| GNL3L Grn1 putative GTPase and GTP-binding protein domain | U6NFF9 | 9 | 2 | 1.25% | 62254.9 | 9.49 |
| Major facilitator superfamily MFS-1 domain containing protein | U6NKQ9 | 9 | 2 | 1.88% | 63960.3 | 6.16 |
| Biotin lipoate A B protein ligase domain containing protein | U6NKY3 | 9 | 2 | 2.64% | 34263.6 | 7.64 |
| Cold-shock protein domain containing protein | U6NMB8 | 9 | 2 | 1.39% | 40095 | 9.25 |
| Acyltransferase ChoActase COT CPT domain containing protein | U6NMS0 | 9 | 2 | 0.79% | 72349.9 | 8.37 |
| C-type lectin domain containing protein | U6NT51 | 9 | 2 | 0.94% | 58573.2 | 6.32 |
| MICOS complex subunit MIC60 | U6PQ62 | 9 | 2 | 0.74% | 75791.6 | 6.33 |
| von Willebrand factor and Endoglin CD105 antigen domain | U6NNY1 | 9 | 2 | 0.74% | 147619 | 4.88 |
| Cation-transporting ATPase | U6NNZ2 | 9 | 2 | 0.50% | 135556 | 7.53 |
| Nuclear receptor NHR-6 | U6NPV7 | 9 | 2 | 1.84% | 36649.8 | 7.35 |
| Serine threonine protein kinase-related domain containing protein | U6NQW1 | 9 | 2 | 1.12% | 70342.9 | 9.6 |
| Patched domain containing protein | U6NRH1 | 9 | 2 | 1.27% | 54261.6 | 6.55 |
| Uncharacterized protein | U6NP07 | 9 | 2 | 1.85% | 36118.3 | 9.27 |
| E3 binding and 2-oxoacid dehydrogenase acyltransferase domain | U6NU08 | 9 | 2 | 1.08% | 51098.1 | 7.65 |
| Major antigen | U6NU99 | 9 | 2 | 1.53% | 51657.2 | 5.11 |
| Uncharacterized protein | U6PKZ8 | 9 | 2 | 3.26% | 24231.1 | 5.67 |
| Endonuclease-reverse transcriptase | U6NUR4 | 9 | 2 | 4.60% | 37040 | 9.4 |
| Uncharacterized protein | U6P0D5 | 9 | 2 | 1.32% | 42745.8 | 8.1 |
| KH domain containing protein | U6NW46 | 9 | 2 | 0.59% | 96106.9 | 7.38 |
| Kinesin domain containing protein | U6NWB1 | 9 | 2 | 0.87% | 130218 | 9.29 |
| Uncharacterized protein | U6NWC2 | 9 | 2 | 1.06% | 119988 | 4.97 |
| Actin-binding domain containing protein | U6NWN5 | 9 | 2 | 1.97% | 40023.5 | 8.05 |
| Ankyrin domain containing protein | U6NQ04 | 9 | 2 | 2.44% | 27034.4 | 6.07 |
| Thyroglobulin type-1 | U6NZE8 | 9 | 2 | 0.29% | 225192 | 6.1 |
| Uncharacterized protein | U6NZT1 | 8 | 2 | 3.40% | 29697.8 | 9.48 |
| Histidine acid phosphatase domain containing protein | U6PDQ8 | 8 | 2 | 1.15% | 48808.4 | 5.67 |
| Uncharacterized protein | U6NW32 | 8 | 2 | 1.04% | 54029.8 | 5.17 |
| Basic helix-loop-helix dimerisation region bHLH domain | U6P4V6 | 8 | 2 | 3.91% | 20757 | 9.58 |
| RUN and RabGAP TBC domain containing protein | U6P4Z3 | 8 | 2 | 0.68% | 101278 | 7.14 |
| BAG family molecular chaperone regulator 2-like | U6P599 | 8 | 2 | 11.36% | 10087.4 | 8.75 |
| Nucleoside diphosphate kinase | U6P6F5 | 8 | 2 | 12.99% | 17466.9 | 7.81 |
| Bicaudal-D protein domain containing protein | U6P7S6 | 8 | 2 | 0.75% | 90442 | 4.99 |
| Nipped-B-like protein | U6P8F1 | 8 | 2 | 0.28% | 245773 | 5.69 |
| DNA mismatch repair protein MutS domain containing protein | U6P8L8 | 8 | 2 | 0.77% | 87882.8 | 7.91 |
| Cullin and Cullin protein domain containing protein | U6P8V8 | 8 | 2 | 0.70% | 115767 | 6.58 |
| Arrestin domain containing protein | W6NEC7 | 8 | 2 | 1.35% | 50487.4 | 7.92 |
| Protein C52E12.1 | U6P8Z4 | 8 | 2 | 0.71% | 94386.2 | 8.85 |
| Adenylyl cyclase class-3 4 guanylyl cyclase domain | U6P9G4 | 8 | 2 | 12.77% | 5592.48 | 9.24 |
| Protein CED-7, isoform b | U6PGF2 | 7 | 2 | 2.21% | 31050.1 | 7.05 |
| Uncharacterized protein | U6PBV3 | 7 | 2 | 0.76% | 87303.3 | 5.67 |
| Major sperm protein domain containing protein | U6PDM2 | 7 | 2 | 0.38% | 210896 | 5.93 |
| Bestrophin domain containing protein | U6PF28 | 7 | 2 | 1.46% | 40260.8 | 5.96 |
| TFIIH p62 subunit and BSD domain containing protein | U6PF34 | 7 | 2 | 0.82% | 70073.3 | 5.21 |
| Vps51 Vps67 domain containing protein | U6PSM2 | 7 | 2 | 0.89% | 62649.8 | 5.19 |
| SCP extracellular domain containing protein | U6PG02 | 7 | 2 | 0.82% | 78237.7 | 8.82 |
| Peptide chain release factor and Class I peptide chain release factor | U6NW67 | 7 | 2 | 1.24% | 45303.1 | 8.59 |
| Uncharacterized protein | U6PHV0 | 7 | 2 | 1.69% | 37014.9 | 9.42 |
| Protein F18A1.7 | U6PL56 | 7 | 2 | 1.76% | 31689.9 | 5.1 |
| Protein TTN-1, isoform d | U6PL57 | 7 | 2 | 0.33% | 173114 | 9.26 |
| Gag-pol polyprotein | U6PNJ0 | 7 | 2 | 1.29% | 43062.4 | 8.25 |
| Major facilitator superfamily MFS-1 domain containing protein | U6PNQ8 | 7 | 2 | 1.23% | 53621.7 | 8.53 |
| Similar to R52.2 | U6PNR9 | 7 | 2 | 5.22% | 13166.1 | 5.18 |
| Cyclin F-box domain containing protein | U6PP54 | 6 | 2 | 1.34% | 75045.6 | 8.86 |
| Proton-dependent oligopeptide transport (POT) protein | U6PPS8 | 6 | 2 | 2.48% | 22840.5 | 6.88 |
| Uncharacterized protein | U6PQ73 | 6 | 2 | 0.48% | 167078 | 8.4 |
| Filamin ABP280 repeat domain containing protein (Fragment) | U6PQJ9 | 6 | 2 | 0.59% | 165211 | 6.33 |
| Speract scavenger receptor domain containing protein | U6PQX6 | 6 | 2 | 0.21% | 323190 | 7.12 |
| Uncharacterized protein | U6PRP1 | 6 | 2 | 1.88% | 31016.7 | 7.53 |
| Neuropilin and tolloid-like protein 1-like | U6PRW1 | 6 | 2 | 10.42% | 10830.2 | 5.65 |
| Translation initiation factor eIF3 subunit domain containing protein | U6PSP8 | 6 | 2 | 4.00% | 22380.8 | 4.78 |
| Diacylglycerol kinase | U6PTL5 | 6 | 2 | 0.63% | 105290 | 6.32 |
| Histone H2A | U6PU35 | 6 | 2 | 7.09% | 13440.4 | 10.7 |
| Tyrosine protein kinase domain containing protein | U6PUM8 | 6 | 2 | 1.26% | 97800.2 | 6.21 |
| Zinc finger domain containing protein | U6PUN6 | 6 | 2 | 0.37% | 180090 | 6.61 |
| Uncharacterized protein | U6PVF5 | 6 | 2 | 2.74% | 33224.2 | 9.91 |
| Histone H2B | U6PVQ2 | 6 | 2 | 7.32% | 13640.6 | 10.43 |
| CRE-RIL-1 protein | U6NXB5 | 6 | 2 | 4.35% | 16331.8 | 9.81 |
| Kinesin-like protein | U6PZ84 | 6 | 2 | 0.86% | 79375.9 | 6.18 |
| Histidine acid phosphatase and ATPase domain containing protein | U6PZA1 | 6 | 2 | 0.71% | 110600 | 5.65 |
| Heat shock protein DnaJ domain containing protein | U6Q0T3 | 6 | 2 | 0.29% | 231187 | 6.34 |
| Caffeine-induced death protein 2 domain containing protein | W6N9U9 | 6 | 2 | 7.14% | 14226.1 | 6.74 |
| Bardet-Biedl syndrome 1 protein isoform 2 | W6NDG1 | 6 | 2 | 3.35% | 26387.2 | 6.2 |
| Uncharacterized protein | U6NL56 | 6 | 2 | 3.52% | 22217.1 | 5.33 |
| Voltage-dependent calcium channel domain containing protein | U6NQV7 | 6 | 2 | 0.85% | 64352.9 | 9.16 |
| Serine threonine protein kinase-related domain containing protein | U6PMR8 | 6 | 2 | 0.38% | 144474 | 9.58 |
| Myosin tail domain containing protein | U6P8T6 | 6 | 2 | 0.88% | 117715 | 5.27 |
| Similar to piggyBac-derived 2 (AGAP012114-PA) | U6P980 | 6 | 2 | 1.88% | 30155.2 | 4.8 |
| Glutamyl-tRNA(Gln) amidotransferase subunit A, mitochondrial | W6NW31 | 6 | 2 | 1.47% | 51937.6 | 5.74 |
