# Supplementary material for: Proteomic analysis revealed T cell hyporesponsiveness induced by Haemonchus contortus excretory and secretory proteins
Source: Vet Res. 2020 May 13;51:65. doi: 10.1186/s13567-020-00790-0 (PMC7222441; doi:10.1186/s13567-020-00790-0)
Supplement: Supplementary file 3 — Additional file 3: Full list of identified T cell binding receptors in vitro. [file 13567_2020_790_MOESM3_ESM.docx]

**Additional file 3: Table S3. Full list of identified T cell binding receptors in vitro.**

| **Proteins description** | **Uniprot ID** | **Peptide count** | **Unique Pep Count** | **Cover Percent (%)** | **MW(Da)** | **PI** |
| --- | --- | --- | --- | --- | --- | --- |
| Albumin (Fragment) | B3VHM9 | 75 | 25 | 36.54% | 66312.12 | 5.58 |
| Complement component 3d (Fragment) | Q207D0 | 13 | 4 | 6.14% | 33174.55 | 7.17 |
| Glyceraldehyd-3-phosphate dehydrogenase (Fragment) | Q8HY38 | 9 | 3 | 3.16% | 20309.07 | 8.51 |
| KiSS-1 metastasis-suppressor | C6KYM7 | 9 | 3 | 4.44% | 14393.42 | 11.23 |
| Kisspeptin | D1MFS2 | 8 | 2 | 4.44% | 14383.38 | 11.23 |
| Guanylate cyclase | H2ET88 | 7 | 2 | 0.48% | 116974.9 | 6.62 |
| DNA meiotic recombinase 1 transcript variant | A0A109XWH9 | 7 | 2 | 1.68% | 32762.1 | 5.52 |
| Clusterin-associated protein 1 | G1DG20 | 7 | 2 | 1.14% | 50310.89 | 4.75 |
| Putative uncharacterized protein | G1DGG5 | 6 | 2 | 1.13% | 58649.93 | 8.33 |
| Cytochrome P450 20A1 | G1DGG8 | 6 | 2 | 1.08% | 52541.11 | 6.14 |
| CAPHI DNA polymerase beta | G1DG82 | 6 | 2 | 3.55% | 15623.8 | 9.77 |
| Titin (Fragment) | I6T2T0 | 6 | 2 | 1.06% | 62781.45 | 5.21 |
| Beta-actin | I6W7A2 | 6 | 2 | 1.87% | 41592.17 | 5.45 |
| Beta-actin (Fragment) | G3ELM1 | 6 | 2 | 4.14% | 18948.58 | 5.27 |
| FasL | X2KPF1 | 6 | 2 | 2.17% | 30937.56 | 9.69 |
